# The role of parenting in developmental trajectories of risk for adolescent substance use: a bioecological systems cascade model

**DOI:** 10.3389/fpsyg.2023.1277419

**Published:** 2023-11-20

**Authors:** Kristine Marceau

**Affiliations:** Department of Human Development and Family Science, Purdue University, West Lafayette, IN, United States

**Keywords:** adolescent substance use, parenting (MeSH), dynamic systems, biobehavioral, development, substance use-specific parenting

## Abstract

Parenting is a key influence and prevention target for adolescent substance use, and changes dramatically in form and function during adolescence. This theoretical synthesis reviews evidence of associations of substance use-specific parenting behaviors, dimensions, and styles with adolescent substance use, and integrates key developmental and family theories (e.g., bioecological, dynamical systems, family systems, developmental cascades) and methodological-conceptual advances to illustrate the complex role that parenting plays for the development of adolescent substance use in combination with child and contextual influences. The resulting bioecological systems cascade model centers the dynamic co-development of parenting and child influences in developmental cascades that lead to more or less risk for adolescent substance use. These trajectories are initiated by intergenerational influences, including genetics, parents’ familial environments, and child-parent attachment. Culture and context influences are a holistic backdrop shaping parent-adolescent trajectories. Parenting is influences are conceptualized as a complex process by which specific parenting behaviors are informed by and accumulate into parenting dimensions which together comprise general parenting styles and are informed by the broader family context. The co-development of parenting and child biobehavioral risk is shaped by both parents and children, including by the genetics and environments they do and do not share. This co-development is dynamic, and developmental transitions of individuals and the family lead to periods of increased lability or variability that can change the longer-term trajectories of children’s risk for substance use. Methodological avenues for future studies to operationalize the model are discussed.

## Introduction

At its core, parenting is a series of behaviors, or ways in which parents act in order to socialize and protect the health and safety of their children. As children grow, the parenting behaviors required to support child health and safety can change dramatically. Adolescence, defined as the developmental period between pubertal onset (a biological marker) and the transition to adulthood (an increasingly blurry sociological marker), is a developmental period of change in parenting (Smetana and Rote, 2019). In adolescence, specific parenting goals and behaviors must evolve to support adolescent autonomy development while mitigating developmentally normative risks that could lead to significant changes in the child’s developmental trajectory. Adolescence is also the beginning of the onset window for substance use. Empirical evidence, mostly from animal models and supported in some longitudinal models of humans, shows that substance use itself can alter the trajectory of brain development including in areas of the brain that can affect behavior (Thorpe et al., 2020), particularly if introduced in adolescence (Lees et al., 2020). This makes prevention or delay of substance use a critical parenting goal during adolescence. As developmental goals become more varied and complex, the same parenting behaviors can promote or discourage different developmental goals at different times (Grusec and Davidov, 2015; Smetana, 2017). Specifically, to achieve prevention or delay of substance use, parents must adopt a new set of parenting behaviors specific to substance use. However, some of the same parenting behaviors intended to curb substance use may work in opposition to the goal of encouraging autonomy development. Changes in parenting do not occur in a vacuum, but rather are inextricably linked to children’s biobehavioral risk and responses, are repeatedly perturbed by developmental transitions, and are continually shaped by intergenerational, cultural and contextual, and family factors.

The present theoretical synthesis begins with brief reviews of the conceptualization and evidence of associations of substance use-specific parenting behaviors, dimensions, and styles with adolescent substance use, and key developmental and family theories. Following this, the first contribution of this paper to the literature is an elaboration of a bioecological systems cascade model of substance use risk. This model describes how developmental forces shape the co-development of parenting and child biobehavioral risk and how this co-development occurs in response to transitions that solidify into larger-scale changes multiple timescales within the context of the family and culture. Next, I provide an overview of key methodological-conceptual advances relevant for understanding the role of parenting in developmental trajectories for adolescent substance use, including key findings from each advance as it relates to parenting and adolescent substance use. Following this, a second contribution of this paper is the presentation of a methodological roadmap integrating methodological-conceptual advances for operationalizing and testing the bioecological systems cascade model.

## Substance use-specific parenting behaviors, dimensions, and styles

Recent theoretical perspectives (Smetana, 2017; Morris et al., 2021; Oropesa Ruiz, 2022) describe three major conceptual layers to parenting. Parenting behaviors are defined as the specific strategies parents use with their children. The ways in which parents engage in specific parenting behaviors are informed by and, through repetition, accumulate into parenting dimensions. Commonly studied parenting dimensions include demandingness, parental control (which can be psychological or behavioral in nature), responsiveness, warmth, or involvement. These dimensions together comprise styles of parenting, which are carried out by specific parenting behaviors (Oropesa Ruiz, 2022).

### Substance use-specific parenting behaviors

Parenting behaviors that are commonly examined and linked to more adolescent use include parents’ own substance use (theoretically linked to adolescent use via modeling and increased availability in the home for parents to use; Trucco, 2020), permissive rules about substance use (Trucco, 2020; Mehanović et al., 2022), and ineffective punishment, including parental harsh discipline (Marceau et al., 2021). Communication about substance use is more nuanced. An integrative review found that conversations about health risks are related to lower substance use, whereas conversations about parents’ own use, permissive messages, and conversations about consequences were related to higher use (Carver et al., 2017).

Parental monitoring, which comprises knowledge of as well as parental control over adolescents’ whereabouts and activities is perhaps the most robustly studied parenting behavior in relation to adolescent substance use. Research has accumulated showing that in early studies, despite being termed “monitoring,” parental *knowledge* was actually measured (Stattin and Kerr, 2000) and further knowledge is primarily gathered through child freely disclosing information (e.g., rather than a parenting behavior; Kerr et al., 2010). In the past couple of decades, increased attention has been given to parent- and child-based sources of knowledge. Parent-based sources of knowledge include parental solicitation—asking children about where they are going, with whom they spend time, and what they are doing, and parental control—creating rules (i.e., curfews; rules about drinking) that limit where, who, and what children are doing. The primary child-based source of knowledge is disclosure—children freely offering information to parents about where they go, with whom, and what they were doing or plan to do, and on the opposite end of the spectrum, secrecy (Lionetti et al., 2019). Links between adolescent secrecy and increased substance use is consistent across cultures (Kapetanovic et al., 2020a).

Although correlated (Keijsers, 2016), sources of parental knowledge are often differentially associated with adolescent substance use. More child disclosure is linked most robustly to less substance use later, though parental solicitation is typically not linked to substance use later, and parental control is only sometimes linked (Stavrinides et al., 2010; McCann et al., 2016; Kapetanovic et al., 2020b). Critically, some aspects of monitoring are not always protective: consistent parental solicitation and control was related to a higher probability of substance use initiation and more severe smoking (Marceau et al., 2020b), and parental solicitation at one assessment predicted increased substance use at the next (Kapetanovic et al., 2020b) and specifically for older adolescents (Berglund et al., 2022). These findings suggest different mechanisms by which child- and parent-driven parental knowledge are associated with substance use, and highlight the critical role of evocative child effects as child-based sources of knowledge are consistently more strongly associated with substance use outcomes than parent-based sources (Keijsers, 2016). Although these results are limited in terms of causal inference, they hint that increasing the consistency of child disclosure may help prevent substance use, but that increasing the consistency (as opposed to responsivity) of parent-based sources of knowledge (i.e., solicitation) could backfire and lead to higher rates of substance use (Marceau et al., 2020b).

### Parenting dimensions

The mixed findings for parent-based sources of knowledge could stem in part from the observation that same parenting behavior can be communicated to the child in a variety of ways and with distinct emotional tones, for example along dimensions of warmth to punitiveness and/or calm to high affect (which could be positive like excitement or negative like fear). The accumulation of the form (i.e., specific behavior), and function (i.e., intended parenting goal), and emotional tone of parenting behaviors together inform or make up overall parenting dimensions. Although demandingness and responsiveness or warmth are highlighted in the literature on parenting styles, additional dimensions have been examined in the literature, such as hostility and involvement. In addition, more relational dimensions (e.g., a reflection or characterization of the parent-child relationship more so than the parent or the child within the relationship), including lower closeness (Amutah-Onukagha et al., 2023) and more conflict (Breivik et al., 2009), have also been linked to substance use (Rusby et al., 2018). As noted above, adolescence comes with unique tensions in balancing parenting goals and opposing behavior—for example, exerting behavioral control to reduce opportunities for substance use could also stifle adolescent autonomy development. This tension and balance is reflected in classical findings of increased conflict and decreased closeness in middle adolescence that resolves later in adolescence (Laursen and Collins, 2009; Marceau et al., 2015b; Xie et al., 2021). In general, more warmth, responsiveness, involvement, and closeness are related to less substance use, whereas more conflict and hostility is related to more substance use (Marceau et al., 2020a; Trucco, 2020).

### Parenting styles

In turn, these dimensions have been reduced into a set of parenting styles. One of the most influential models of parenting as it relates to the development of adolescent substance use is Baumrind’s conceptualization of parenting styles (Baumrind, 1966), later modified by Maccoby and Martin (1983). In current conceptualizations, two dimensions of parenting—responsiveness (also operationalized as warmth or involvement) and demandingness (also operationalized as control) are set along two axes, creating four quadrants reflecting distinct styles of parenting: *authoritative*, high demands (specifically behavioral control) and high responsiveness, *authoritarian*, high demands (specifically psychological control) and low responsiveness, *permissive or indulgent*, low demands and high responsiveness and *neglectful*, low demands and low responsiveness. Generally, authoritative parenting styles are protective against substance use, although indulgent styles may also confer less risk than authoritative and neglectful styles (Calafat et al., 2014). A complementary model to considering parenting styles as aggregates of dimensions that exert main effects on child behavior, Darling and Steinberg (1993) proposed a more integrated approach, highlighting how parenting styles act as more of a backdrop or context for which different parenting practices are related to child outcomes.

## Key theoretical frameworks for parenting and adolescent substance use

When examining the role of parenting for adolescent substance use, parenting is often placed in the microsystem of Bronfenbrenner’s bioecological model, used as a theoretical backdrop to studies of individual differences. This dovetails with a broader view of the development of adolescent substance use, which is often conceptualized in terms of developmental cascades including multiple risk factors across multiple domains (genetic, prenatal, parenting, peers, neighborhoods, stress, child characteristics) that accumulate across development (Dodge et al., 2009; Marceau et al., 2021). Recent developmental perspectives on adolescent substance use have placed individual developmental cascades within the context of the ecological systems framework (Trucco and Hartmann, 2021). These advances in developmental models have occurred simultaneously and yet somewhat apart from advances in family theories and methodologies.

Family systems theory highlights that the parent-adolescent relationship is only one specific sub-system within a family—the family system often includes marital relationships, sibling relationships, multiple parent-child relationships, and extended kinships (Cox and Paley, 2003). These dyadic sub-systems are not independent—hostility and conflict in the marital relationship for example can spill over to cause hostility in the parent-adolescent relationship, or can lead to alliances, enmeshment, or compensatory behaviors in parent-adolescent relationships (Cox and Paley, 2003). Advances in family theory have underscored the importance of considering multiple family relationships for understanding associations of parenting and adolescent substance use (Neiderhiser et al., 2013; Xia et al., 2020).

The dynamic systems meta-theory is a theoretical orientation that conceptualizes development in any domain as a series of more stable structural patterns and phase transitions that move the system into a new stable structure (Witherington, 2007). Applied to parent-adolescent dyads, stable patterns of interaction in middle childhood are disrupted by transitions around the start of adolescence (e.g., puberty, school transitions) which lead to increased variability in the interactional patterns between parents and youth that foreshadow phase shifts in relationship quality (Granic et al., 2003). This theory has been largely supported over the past few decades, mainly regarding emotional dynamics. For example, Branje (2018) highlights the role of emotional variability during conflict interactions to shape the trajectory of mothers’ control and adolescents’ disclosure. Lougheed (2020) describes parent-adolescent dyads as temporal interpersonal emotion systems, whereby individual interactions allow parents and youth to co-construct the emotional tone of the relationship, and repeated interactions co-constructing similar emotional tones and interaction patterns (i.e., responses to the others’ emotions, behaviors, and words) stabilize over days, weeks, and years.

Dynamic systems models have been linked to antisocial behavior (Granic and Patterson, 2006), a strong predictor of adolescent substance use, though not yet explicitly to adolescent substance use outcomes. Although dynamic systems theories are complementary to family systems theories, in adolescence most work using dynamic systems perspectives is specific to mother-adolescent dyads’ emotional tone during interactions. Applying dynamic family systems approaches to examine multiple parents and the substance use of multiple children in families is a critically important future direction.

## A bioecological systems cascade model

**FIGURE 1:**
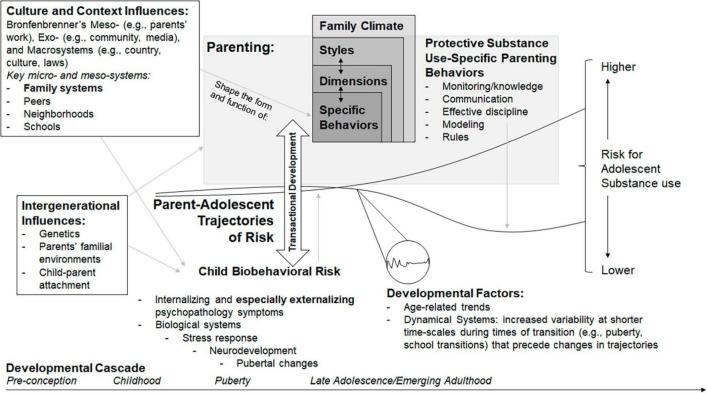
A bioecological systems cascade model for the role of parenting in developmental trajectories of risk for adolescent substance use. Developmental cascades of risk are conceptualized as dynamic co-development shaped by transactional processes between child biobehavioral risks (center, **bottom**) and parenting influences (center, **top**). Parenting risk includes substance use-specific parenting behaviors, which both inform parenting dimensions and are shaped by them, just as parenting dimensions inform and are shaped by parenting styles, contextualized by the family climate. The two lines in the center represent two exemplar individual parent-adolescent trajectories across childhood and adolescence culminating in late adolescent/emerging adulthood substance use risk (right side of the figure). These exemplar trajectories represent the longer-term age-related trends captured by developmental cascades of risk that unfold over time (timeline depicted across the bottom of the figure). In the lower left, intergenerational influences are conceptualized as “starting points” for developmental cascades of individual trajectories of risk. Parentadolescent developmental cascades (indeed the entirety of the model) are shaped by cultural and contextual influences (**top**, left). The course of each individual parent-adolescent trajectory is further influenced by developmental factors (**bottom** right), including shorter-term periods of increased variability induced by individual (child or parent) and family transitions which have the capacity to alter longer-term developmental trajectories (**bottom**, right).

Figure 1 depicts a theoretical integration of the conceptualization of parenting behaviors, dimensions, and styles with ecological systems, developmental cascade, and family and dynamic systems models as they relate to the development of adolescent substance use. Briefly, this integration produces a bioecological systems cascade model that centers the dynamic co-development of parenting and child influences within parent-adolescent developmental cascades that lead to more or less risk for adolescent substance use. Parent-adolescent developmental cascades are initiated by intergenerational influences, including genetics, parents’ familial environments, and child-parent attachment and, drawing on ecological models, shaped by culture and context influences. Parenting and child biobehavioral risk are conceptualized as dynamic and co-developing. Child biobehavioral risk includes both psychological/behavioral (e.g., psychopathology) and biological (e.g., stress response, neurodevelopment, puberty) components. Parenting influences are described by the three layers reviewed above, whereby specific (including substance-use specific) parenting behaviors are informed by and form parenting dimensions and arise in general parenting styles which, drawing from family systems theory, are also informed by the broader family context. Incorporating the dynamic systems frame, adolescents’ developmental transitions (e.g., school transitions, puberty) lead to periods of increased lability or variability in parent-child relationships that can change the longer-term trajectories of adolescents’ risk for substance use. Integrating family systems theory, developmental transitions of the family are expected to lead to similar periods of increased lability or variability that too can catalyze longer-term trajectory changes.

### Parent-adolescent trajectories of risk

In the center of Figure 1, parent-adolescent trajectories of risk are conceptualized in line with developmental cascades models. Although to a large extent development is stochastic (i.e., partially random, and probabilistic rather than deterministic), it is hypothesized that developmental forces that shape trajectories in a way that makes some developmental trajectories more likely than others. These developmental forces can be conceptualized in terms of constraints (forces that funnel individuals and families toward more narrow or canalized developmental trajectories, like when two rivers meet and form one) and catalysts (divergent forces that open new developmental trajectory possibilities, like an obstruction that can lead to the branching of a river into multiple streams). Developmental cascades to substance use are therefore described by diverse trajectories of risk (e.g., Trucco et al., 2016, 2018; Zucker et al., 2016; Marceau et al., 2020a,^2021^), which begin to differentiate and increase (in terms of risk for substance use) for some around puberty, and result in varying levels of risk for adolescent substance at the peak time of risk in late adolescence and early adulthood. In the bioecological systems cascade model, developmental cascades do not only describe adolescent risk trajectories (as in most studies of developmental cascades), but rather developmental cascades reflect the co-developing trajectories of parent- and child/adolescent- risk behaviors for substance use.

### Intergenerational influences and culture and context

Depicted on the left-hand side of Figure 1, key developmental constraint forces that can act as funnels at the earliest stages parent-adolescent developmental cascades of substance use risk include intergenerational influences and cultural and contextual influences. Intergenerational influences include genetic and environmental influences (e.g., McGue et al., 2014; Jami et al., 2021; Sally et al., 2022), which may be transmitted in part via behavioral genetic mechanisms (reviewed below) and early child-parent attachment (Schindler, 2019). These factors are expected to influence child biobehavioral risk but also the parenting context, and critically, their co-development (described in the section “Transactional development”). Culture and contextual influences (Chassin et al., 2019; Rothenberg et al., 2020; Lansford, 2022) include many of the classical distal influences including country, culture laws, community, media, and parents’ work environments. For example, some communities and/or religions generally discourage alcohol and other substance use, and families in those communities would begin at reduced risk for adolescent substance use. Key micro- and meso-systems highlighted in the literature to date, include schools, neighborhoods, peers, and especially the family system, see Trucco (2020) for review.

Culture and context influences can operate as early constraints setting initial developmental trajectories and can continue to shape development over time, for example via a social control/opportunity mechanism (Shanahan and Hofer, 2005). Socio-cultural environments (e.g., the key micro- meso-systems as well as classic distal influences highlighted above) that are associated with more social control (e.g., stronger social norms around not using substances, or higher social sanctions for using) can act as constraints that reduce the expression of (a) youths’ genetically informed predispositions (an intergenerational influence) to engage in substance use and/or (b) parents’ choices surrounding socially accepted substance-specific parenting. On the other hand, socio-cultural environments that promote individual differences and/or have lower social control can act as catalysts of divergent trajectories that allow for greater expression of youths’ genetically informed predispositions to engage in substance use and/or parents’ parenting choices. In line with this model, gene-environment interaction work has shown that these types of cultural influences can serve as a protective factor and suppress the expression of individual differences and genetic influences that would lead to more risk for adolescent substance use (Barr and Dick, 2020). These cultural factors also shape the form and function of substance use-specific parenting behaviors, for example by informing parents’ own views via social norms, rewards, and sanctions on for example, appropriate discipline or monitoring strategies (Lansford, 2022). Cultural normativeness may also influence how parents and children interpret parenting behaviors, and thus moderate or shape transactional developmental influences between child and parent behaviors (Lansford, 2022).

### Transactional development

Integrating a developmental perspective grounded in dynamic systems, across childhood and adolescence, transactional influences between child behaviors and parenting behaviors and context are expected to shape parent-adolescent trajectories of risk (depicted by the bidirectional arrow at the center of Figure 1). In line with dynamic systems theories, increased variability in parenting and adolescent behaviors and emotional tenor at major transitions (pubertal milestones, school transitions) and smaller transitions (e.g., breaks from routines such as the start and end of school breaks, shifting extracurricular schedules) open opportunities for re-balancing in the parent-adolescent dyad (e.g., bottom right corner of Figure 1; Hollenstein and Lougheed, 2013; Lougheed, 2020).

For example, family shifts in routine like transitioning from the school year to a summer schedule may produce changes in the amount of time families spend together and the topics of conversation. A family that becomes less structured during the summer may find that youth have more free time and more autonomy in choosing which friends they see and what they do together—including increased opportunity for substance use. Parents can embrace the relaxed structure, thereby being less restrictive (which may allow more autonomy development) or can respond by imposing more structure or oversight/monitoring to protect the youth from dangerous situations or substance use engagement. Depending on the needs of the adolescent, either choice could lead to adolescent-parent interactions that are more positive (adolescents appreciating more relaxed time or thriving on more structure) or negative (adolescents making poor choices that elicit consequences or pushing back against oversight).

For another family, the same transition from school to summer may lead to a different but equally structured schedule, with parents driving (e.g., by selecting care options) where their children are and what they are doing, for example. This family may experience more daily conflict over differences in opinion between how scheduled the adolescent would like to be vs. what the parent has decided. That increase in daily conflict across many days or weeks may then solidify into a new interactions style that itself promotes disengagement from the family and increases risk for substance use (although this will not necessarily be the case given the stochasticity of development).

In both hypothetical family situations, navigation of changing circumstances opens a window of opportunity for change in parenting behaviors and child biobehavioral risk through shorter-term changes in parent-adolescent interaction patterns that together have the potential to shift substance use risk trajectories. Embedded in a family systems frame, transitions need not even apply directly to the focal adolescent. That is, changes experienced by siblings and parents (e.g., work, marital transitions) would also be expected to increase variability in parent-adolescent behaviors, emotions and relationship quality dimensions in daily interactions that have the potential to canalize into new interactional patterns that push youth and parents towards or away from risker trajectories with regard to adolescent substance use.

#### Child biobehavioral risk

A core mechanism of transactional processes between parents and youth is child evocative effects, which underscore that children to some extent evoke the parenting that they receive, in part through their genetically influenced behaviors (Neiderhiser et al., 2004, 2007) in ways that change over time (Marceau et al., 2016). Typically, studies focus on youth internalizing and externalizing symptoms for capturing the child’s behavioral risk for substance use. Psychopathology symptoms are influenced by intergenerational, cultural and contextual influences including genetics (King et al., 2004; Elkins et al., 2007), and have been shown to evoke parenting behaviors in part through adolescents’ genetic influences (Marceau et al., 2013).

The bioecological systems cascade model also highlights the importance of including additional key biological systems identified in the literature, including neurodevelopment (e.g., Koyama et al., 2017), stress response (e.g., Marceau and Abel, 2018), and pubertal changes (e.g., Marceau and Jackson, 2017; Marceau et al., 2019, 2021) for understanding how children’s biobehavioral development shape parent-adolescent trajectories of risk for substance use (bottom of Figure 1). For example, youth who go through puberty (a biological but also social transition) at an earlier age may be viewed as older due to their physical appearance, and thus parents may be more likely to relax rules about drinking (and generally treat their child more like an adult) which can lead to increased risk of substance use (e.g., Bucci et al., 2021). This parental response to a biological transition in children may be driven by parents’ perceptions and/or youths’ advocation for more mature treatment (social responses). However, because brain maturation and pubertal development do not necessarily occur simultaneously (e.g., some but not all components of brain development are driven by puberty; Herting and Sowell, 2017), these changes in substance use-specific parenting, if moving toward more permissive when youth are not yet ready for more autonomy, can be particularly harmful.

#### Parenting

The role of parenting includes substance use-specific parenting behaviors, which both inform parenting dimensions and are shaped by them, just as parenting dimensions inform and are shaped by parenting styles. Substance use-specific parenting protective factors are expected to mediate the parenting context, influence, or to *be* the specific parenting mechanisms most proximally related to adolescent substance use. Further, parenting is expected to change during transitions as described above, and as adolescent behavior changes. That is, youths’ progression to a next milestone of risk for substance use (e.g., from biobehavioral risk to initiation, regular use, heavy/problematic use, and substance use disorder; Jackson et al., 2021), especially when known by the parent, may also serve as a transition that catalyzes divergence or opens new possibilities of parent-adolescent trajectories of risk depending on how the family responds. For example, parents may respond to their adolescent being caught initiating alcohol with increases in their protective substance-use specific parenting behavior. The adolescent may then either respond positively and not progress toward more substance use with minimal changes to the parent-adolescent relationship or react negatively to these new autonomy-restricting parenting behaviors with hostility in daily conflicts and conversations. In this latter case, and particularly if adolescent hostility is met by parent hostility in return, the new more hostile interaction patterns during repeated parent-adolescent interactions can become canalized into a new interaction schema enforced by both members of the dyad that changes where the dyad falls in terms of parenting dimensions (e.g., increased hostility) and styles. As the adolescent progresses to more severe substance use, again parents may change their behaviors and choices to, for example, a stricter stance to try to mitigate risk or to a more permissive stance to try to repair the relationship. Each parenting choice would elicit a different response from the adolescent depending on the intergenerational, cultural, and family context which would have the opportunity to further shift trajectories of risk.

Because of the critical role of the family system for a single dyad, in this case the parent-child dyad, these behaviors are positioned within the context of the family climate. Supports, strains, and changes in other family dyads could also inform parental and adolescent responses to each other. In general, in supportive broader family contexts, the family context may buffer against any harmful effects of increased conflict or decreased closeness that occur during transition points and changes in parenting and child biobehavioral risk. However, in stressful broader family contexts, parents and youth may have fewer resources to draw on to cope with these changes, which could exacerbate the speed and severity with which the relationship and parent-adolescent trajectories shift.

In summary, according to the bioecological systems cascade model, parenting and child biobehavioral risk are constantly co-developing, with more variability in the short-term due to any number of transitions faced by families leading to slightly altered trajectories in the medium term that can canalize into larger differences in the long-term, and in the end-point of parent-adolescent trajectories of adolescent substance use risk. This model emphasizes individual differences in parent-adolescent dyads and the critical role of contexts and transitions for continually altering trajectories of risk. This model also helps to explain why the literature mapping developmental trajectories of adolescent substance use via mediation and moderation analyses is so fraught with mixed findings: Most studies examine sample averages without addressing variability in trajectories and contexts. In order to successfully test these ideas, the integration of advanced study designs and statistical methods are required. Recent advances provide important tools for testing this integrative model, reviewed below.

## Methodological-conceptual advances

### Within-family processes and between-family differences

One of the major advances in the study of parenting and adolescent development is attention to within-family vs. between-family processes. Within-family variation captures lability (variability or fluctuations) and developmental processes within a single family and can only be captured via repeated measures within families over time. Between-family variation, on the other hand, describes more stable differences from one family to another rather than changes within a family. Conceptually, parenting styles have been interpreted more as a stable characteristic of parents, and thus are likely best captured in terms of between-family differences.

Parenting dimensions, on the other hand, have been the subject of much longitudinal research. A growing body of literature has shown developmental trends as well as lability, or fluctuations over months or years at the within-family level, of parenting dimensions (Marceau et al., 2015b; Lippold et al., 2018; Zheng and McMahon, 2022) and behaviors (e.g., parental knowledge and solicitation; Keijsers, 2016; Lippold et al., 2016; Marceau et al., 2020b). These studies have ultimately focused on understanding between-family differences in how within-family processes are related to substance use. That is, by examining repeated measures of a parenting dimension or behavior via multilevel models of change or latent growth curve modeling, researchers can obtain an intercept score, a slope score, and a series of residual scores. The range (or standard deviation) of those residual scores conceptually index lability, or how dramatic the fluctuations over time in the parenting dimension or behavior are, after accounting for the families’ longer-term developmental trajectory (Marceau et al., 2015b). Using these scores, investigators can answer questions related to longer-term trajectories like “Are the families with steeper declines in parental knowledge over time the same families that have adolescents with more substance use, compared to families with less steep declines in parental knowledge over time?” and “Do families with higher within-family fluctuations in parental knowledge over time have adolescents with more substance use compared to families with less within-family fluctuations in parental knowledge over time?” This approach has largely found support for within-family processes (both longer-term developmental change and lability in warmth, hostility, and aspects of parental monitoring) being associated with adolescence substance use (Lippold et al., 2016, 2018; Marceau et al., 2020b).

Using this methodology, investigators can also ask questions related to core features of the bioecological systems cascade model, including the parent-child dynamics at transitions in novel ways, for example: “Do families with youth who are in the middle of puberty have higher within-family fluctuations in parental knowledge over time than families with youth who are pre-puberty?” This approach can be extended in the future to index changes and lability in other key components of parenting (e.g., substance use-specific parenting behaviors, parenting styles) over time in addition to the dimensions that have been studied thus far.

Finally, within- and between- person processes have been directly tested via the use of random-intercept cross-lagged panel models (and other similar advanced panel model techniques capable of separating within- and between-family processes; see Orth et al., 2021 for a review). These models have been used to robustly examine associations of parenting dimensions and behaviors with child behavior that are known risks for substance use (e.g., Lougheed et al., 2022), albeit with far fewer investigations for substance use outcomes. One recent study yielded evidence of some between- and some within-family contribution to associations of parenting dimensions and behaviors with adolescent substance use (Robillard et al., 2022). Further application of these models to substance-specific parenting behaviors at multiple timescales will help to identify the hypothesized transactional developmental processes between child characteristics and parenting over time, and the timescale(s) in which they operate.

### Variable-centered and person-centered approaches

Applied to parenting, variable-centered methods focus on individuals’ levels of parenting styles, dimensions, and behaviors, implemented through observed or constructed scores, or latent variable modeling. These variables may then be used in, for example, developmental cascade models predicting substance use (Dodge et al., 2009; Marceau et al., 2020a,^2021^; Trucco and Hartmann, 2021). Or, parenting dimensions or levels can be dichotomized based on some theoretically meaningful cut-off and aggregated into typologies, as often done to create the classical parenting styles. An advantage to this theory-driven typology approach is that theoretically meaningful groups or dimensions are created that are similar across studies (as they are researcher-imposed). However, the groups may not reflect the sample or population well, and the number of variables and thresholds for each variable that can be considered at once is limited (as the number of groups exponentially increases with each variable added). Thus, there is a balance between theoretically meaningful groups and creating groups that can be populated by enough people in the sample that they are useful. Using dimensional variables, there may be floor or ceiling effects (as is often the case for survey measures of self-reported parental warmth, for example).

Person- (or family-) centered approaches, on the other hand, group individuals based on the co-occurrence of measured parenting dimensions or behaviors, typical for latent class analysis (using categorical variables) or latent profile analysis (using continuous variables). The data-driven nature of person-centered approaches carries strengths and weaknesses. An advantage to this approach is that novel insights can arise for understanding how different parenting dimensions and behaviors co-occur in a sample. The groups can also be formed using many different variables, allowing for complex interactions among variables in multidimensional space to be captured, which may be particularly useful for more comprehensive measures of parenting in the family context. For example, these approaches have been proposed a way to view complex interactions (Ennett et al., 2016), and examine the co-occurring patterns of parenting behaviors and dimensions to form typologies which may or may not map on to traditional parenting styles. However, the results from these person-centered approaches are specific to the variables included in the analysis and the sample analyzed leading to substantial challenges in interpreting and replicating findings across studies and suffer greater generalizability and robustness challenges with smaller samples sizes.

Person-centered approaches have been leveraged to form groups on a wide array of specific family and parenting-related variables to understand how combinations of risk factors are associated with adolescent substance use. For example, studies have included measures of family functioning (Cordova et al., 2014; Rojas et al., 2023), family routines (e.g., frequency per week of family dinners, housework, fun activities, and religious activities; Abar et al., 2020), and family structure and timing of family structure transitions (Johnston et al., 2020). Others have focused on parenting dimensions specifically, such as hostility and warmth dimensions across three family dyads (Xia et al., 2020), and closeness to resident and non-resident parents in stepfather families (Amato et al., 2016). A recent study examined multiple parenting behaviors specifically regarding media parenting (e.g., device access, communication and monitoring of online activities; Cox et al., 2021). A set of studies focused specifically on communication about substance use, incorporating parent-based and family communication styles (Choi et al., 2017), topics and frequency of parent-teen communication about substance use (Abar et al., 2011; Koning et al., 2012), and adolescent disclosure, secrecy, and lying (Baudat et al., 2022). Across nearly all the disparate family based latent profiles, higher risk profiles emerged from combinations of known risk factors that were linked to substance use in expected directions.

Table 1 reviews studies that have included parenting dimensions that theoretically underlie parenting styles (e.g., responsiveness, demandingness, monitoring, involvement, discipline), sometimes alongside substance use-specific parenting behaviors, in relation to adolescent substance use outcomes. While some studies found empirically derived profiles that were mostly similar to parenting styles and were linked to substance use outcomes in expected directions (e.g., Luyckx et al., 2011; LoBraico et al., 2020), others found profiles that did not match precisely the classical parenting styles and did not link to substance use (e.g., Young et al., 2011; Dembo et al., 2015). Several studies included substance-use specific parenting behaviors in addition to more classical parenting dimensions, yielding groups that also differed in terms of pro- vs. anti-alcohol sentiments by parents, with pro-alcohol stances (e.g., more permissive communication, more parental alcohol use modeling, more approval of adolescent substance use) associated with more substance use (Abar, 2012; Abar et al., 2014; Varvil-Weld et al., 2014).

**TABLE 1:** Literature review of latent profile and class analyses examining aggregate effects of parenting variables in relation to substance use.

References	Notes	*N*	Age	Dimensions (inputs)	Groups	Group description	Associations with substance use
Luyckx et al., 2011	Examined parenting trajectories	1,049	12 annual assessments from age 6 to 18	Parent-reported monitoring, positive parenting, and parental discipline	Indulgent (19%)	Moderate, decreasing monitoring; high, decreasing positive parenting; high increasing inconsistent discipline	Indulgent and especially Uninvolved had increases in alcohol and cigarette use over time
					Uninvolved (17%)	Low, decreasing monitoring; low, decreasing positive parenting; high, stable inconsistent discipline	
					Authoritarian (29%)	High, decreasing monitoring; low, decreasing positive parenting; low, stable inconsistent discipline	
					Authoritative (36%)	High monitoring; high positive parenting; low stable inconsistent discipline	
LoBraico et al., 2020	Family based intervention (control group only)	5,300	6th grade	Adolescent-reported family conflict, positive family climate, parental involvement, effective discipline, parental knowledge, and adolescent positive engagement	Coercive (15%)	High family conflict; low positive family climate, parental involvement, effective discipline, adolescent positive engagement, and parental knowledge	Coercive >Disengaged and Permissive >High functioning for drunkenness frequency
					Disengaged (41%)	Low positive family climate, parental involvement, adolescent positive engagement, and parental knowledge
					Permissive (11%)	High parental involvement, adolescent positive engagement, parental knowledge, and family conflict; low effective discipline
					High functioning (34%)	High positive family climate, parental involvement, effective discipline, adolescent positive engagement, parental knowledge; low family conflict
Abar, 2012		1,143	First-year university students	Adolescents’ retrospective reports of mothers’ and fathers; alcohol modeling, perceived parent approval of alcohol use, alcohol communications, parental monitoring and knowledge, parental trust and support, parental access, and mother/father-teen conflict	High quality (2012: 19%; 2014: 14%)	High parental trust and support, access, alcohol communications, low mother-teen and father-teen conflict	Pro-alcohol linked to the most alcohol use in college (Abar, 2012); pro-alcohol parenting profile had the highest baseline and steepest increases in drinking across several phenotypes (Abar et al., 2014)
					High monitoring (2012: 31%; 2014: 35%)	High parental monitoring and knowledge, relatively higher communication about alcohol, lower approval of student drinking	
Abar et al., 2014		285	Longitudinal subset of Abar, 2012		Anti-alcohol (2012: 30%; 2014: 31%)	Low maternal and paternal alcohol modeling, parental approval of alcohol use, monitoring knowledge, trust and support, access and communication; high perceived mother-teen and father-teen conflict	
					Pro-alcohol (2012: 21%; 2014: 21%)	Heavier maternal and paternal alcohol use, higher parental approval of alcohol use and parent-teen conflict; low monitoring, knowledge, trust and support	
Varvil-Weld et al., 2012		370	Incoming first year college students who enrolled in an intervention (control group only)	Student-reported perceived positive communication, negative communication, monitoring, approval of alcohol use, and parent alcohol use	Positive + pro-alcohol (37.8%)	High positive communication and monitoring, parent approval of alcohol use, and parent alcohol use; low negative communication	Positive + pro-alcohol use had the highest pre-college drinking, and were most likely to be in the high-risk consequences subset
					Positive + anti-alcohol (34.6%)	high positive communication; low negative communication, parent approval of alcohol use, and parent alcohol use	
					Negative mother (19.5%)	Lower positive communication with mothers, parental monitoring; higher negative communication with mothers	
					Negative father (8.1%)	Lower positive communication with fathers, monitoring; high negative communication with fathers, father alcohol use	
Varvil-Weld et al., 2014	Used same measures and methods as Varvil-Weld et al., 2012	1,900	Incoming first year college students who enrolled in an intervention, includes the subsample reported in Varvil-Weld et al., 2012	Student-reported perceived positive communication, negative communication, monitoring, approval of alcohol use, and parent alcohol use	Positive + pro-alcohol (38.2%)	High positive communication and monitoring, parent approval of alcohol use, and parent alcohol use; low negative communication	Intervention to reduce alcohol use was more effective for college students in the positive + anti-alcohol and negative father groups
					Positive + anti-alcohol (34.9%)	High positive communication; low negative communication, parent approval of alcohol use, and parent alcohol use	
					Negative mother (16.5%)	Lower positive communication with mothers, parental monitoring; higher negative communication with mothers	
					Negative father (10.4%)	Lower positive communication with fathers, monitoring; high negative communication with fathers, father alcohol use	
Ennett et al., 2016		1,530	13 year olds	Mothers’ attitude toward adolescent alcohol use, communication about negative consequences of alcohol use, permissiveness, and perceived ease of accessibility of alcohol at home, mothers’ and fathers’ alcohol use, and parenting style dimensions of responsiveness and demandingness	Conservative socialization (53.01%)	Mothers report 3+ messages about negative consequences of alcohol, No permissive messages; low access to alcohol at home, approval of use, and parental use; high parental demandingness and responsiveness	Alcohol use increasing more across grades 6 through 10 in the tolerant groups compared to the conservative groups
					Conservative + low-authoritative (11.57%)	Lower demandingness and responsiveness, higher parent alcohol problems, single-parent households	
					Tolerant + low parental use (29.15%)	High permissive alcohol messaging, access to alcohol at home, demandingness and responsiveness; low negative messages about alcohol, parent alcohol use, approval of Adolescent drinking, White, higher educated	
					Tolerant + high parental use (6.26%)	High parent alcohol use and alcohol problems, slightly less permissive alcohol messaging than tolerant + low use, high demandingness and responsiveness, White	
Dembo et al., 2015	Drug-involved truant youth	190	11–15 years old	Parent involvement, positive parenting, poor monitoring/supervision, inconsistent discipline, and corporal punishment	Low involvement + low positivity (21%)	Lower involvement, positive parenting	No associations with substance use
					High involvement + high positivity (61%)	Lower corporal punishment, higher involvement and positive parenting	
					Uses corporal punishment (18%)	Higher corporal punishment	
Young et al., 2011		1,700	11–15 years old	Parental Bonding Instrument (8 items)	Optimal parenting (20%)	Most helped, loved (tied with typical group), understood, allowed to make decisions and do things they like, likely to be made to feel better; least often ‘treated like a baby’ (tied with typical group)	No associations with substance use
					Typical parenting (54%)	Second highest scores on positive indicators;	
					Moderate parenting (23%)	Third highest scores on positive indicators, least controlled	
					Neglectful and controlling parenting (3%)	Least helped, loved, understood, allowed to do things they like or make decisions, or made to feel better; most often ‘treated like a baby’	

These studies are the result of a PUBMED query on 5/22/23: (“latent profile” or “latent class”) AND (parenting OR parent-child OR family) AND (adolescen*) AND (“substance use” OR “alcohol” OR “drug” OR “tobacco” OR “marijuana” OR “cannabis”). The search yielded 409 Results. Review of titles and abstracts yielded 52 articles that used latent profile analysis or latent class analysis where the indicator variables included parenting variables, and associations with adolescent substance use were presented. Review of full texts then yielded this final set of 9 articles that included exclusively parenting behaviors and dimensions (as opposed to parenting behaviors alongside other risk and protective factors such as peer characteristics, sociodemographic characteristics, etc.) and associations of these classes with substance use outcomes.

It is important to note that this literature review was restricted to studies conducting latent profile analysis of parenting and family behaviors. A growing body of literature has used similar person-specific strategies to place multiple cultural and contextual factors along with parenting in profile analyses to understand broader risks for adolescent substance use (e.g., from an Bronfenbrenner’s ecological systems perspective). Although these person-centered approaches show great promise for understanding how parenting behaviors, dimensions, and styles are related within-individuals/families and jointly influence behavior, thus far the vast majority of these studies are cross-sectional in nature (see Cordova et al., 2014 for an exception).

Future directions that merge advances in within and between- sources of variance and person-centered approaches will be able to address more complex developmental questions relevant to the bioecological systems cascade model. For example, combining these lines of research [e.g., using latent transition analysis (Lanza et al., 2010) or repeated measures of latent profiles/classes in other developmental models], researchers can ask questions such as “Are there profiles of parenting behaviors over time, when considering intercepts, slopes, and lability of multiple parenting behaviors and dimensions, that put youth at increased risk for substance use?” Second, a critical extension of this approach for testing the bioecological systems cascade model is to incorporate both parenting and child biobehavioral risk factors to yield family based profiles that capture transactional development to best inform substance use risk trajectories. Longitudinal extensions including both parent and child biobehavioral risk when applied to the interaction level (e.g., using observed data during conversation tasks or daily diary measures) could yield insights about whether and how dyadic profiles differ and develop across timescales.

### Behavioral genetic approach

A key limitation of the parenting work reviewed thus far is the frequent omission of an individuals’ genetic inheritance (which can change in expression over time) in studies seeking to understand the role of parenting for substance use development (Marceau et al., 2020a,^2021^). A meta-analysis of multiple genetically informed designs using a variety of measures (e.g., observer ratings, parent and youth reports on several different instruments) found that parenting dimensions of warmth, control, and negativity are influenced children’s and parents’ genes as well as environments (except parents’ genes did not contribute to their use of control; Klahr and Burt, 2014). They also found that children’s shared environments contribute to the parenting they receive, consistent with the common interpretation of parenting effects as environmental influences in non-genetically informed literature. In addition, genetic influences play an important role in the intergenerational transmission (Jami et al., 2021) and development of substance use (Hicks et al., 2011; Deak and Johnson, 2021; Lopez-Leon et al., 2021). Relatedly, there are marked differences in parenting when parents are suffering substance use disorders (Chassin et al., 2019). This evidence suggests that parenting influences on adolescent substance use are at least in part confounded by genes that parents and adolescents share.

Inherited genetic risk for substance use likely explains much of the correlation between parent substance use and adolescent substance use (as opposed to parent modeling or substance availability mechanisms; Jami et al., 2021). However, for other parenting behaviors, genetically informed research has yielded findings that suggest a combination of evocative gene-environment correlations (where parenting behaviors are elicited by the adolescents’ genetically informed substance use or behavioral risk for substance use like externalizing problems), and direct environmental influences consistent with the predominant interpretation of parenting effects in the literature (Marceau et al., 2013, 2015a; Jami et al., 2021). Genetic correlations between monitoring and substance use indicating possible evocative child effects (Neiderhiser et al., 2013; Olivares et al., 2016) as well as environmental effects (after controlling for genetic influences; Neiderhiser et al., 2013; Samek et al., 2015).

Evidence from behavioral genetic studies call into question the interpretation of findings from studies that have examined parents’ psychopathology and/or substance use in latent profile analyses. These studies generally have found that groups were differentiated by types and comorbidities of psychopathology, and more severe and more comorbid profiles tend to be associated with more substance use (Flouri and Ioakeimidi, 2018; Lowthian et al., 2020; Essau and de la Torre-Luque, 2021; Burdzovic Andreas et al., 2022). These studies typically conclude that parents’ psychopathology is an environmental risk factor, when the groups are likely informed by both genetic and family environmental influences. It is important to note that these findings are consistent with intergenerational transmission of psychopathology and substance use models, but as they are not genetically informed, cannot speak to the mechanisms of transmission. In the future, it will be important to conduct this work in genetically informed designs (e.g., twin studies, adoption studies, studies including measured genetic variants) that can help to disentangle mechanisms of influence.

## Integrating methods to test bioecological systems cascade models

This integrative review describes a bioecological systems cascade model for the role of parenting in developmental trajectories of risk for adolescent substance use. This model integrates bioecological frameworks by highlighting the role of culture and context influences for shaping parenting, child biobehavioral risk, and their transactional development. This transactional development is informed by intergenerational influences, including genetics, parents’ familial environments, and child-parent attachment, which are conceptualized as laying the groundwork, or setting the start-point for each parent-adolescent’s developmental trajectory. Analytically, these intergenerational influences would best be conceptualized as an early influence beginning developmental cascades. Culture and context influences are best characterized as a holistic backdrop that may shape parent-adolescent trajectories, and analytically would best be conceptualized as a moderator of developmental trajectories. Further, testing portions or the whole of this bioecological systems cascade model would be much stronger if accounting for shared genetic influences and by demonstrating when specific influences operate via gene-environment correlations, by using genetically informed designs.

Parenting influences are conceptualized as a complex process by which specific parenting behaviors are informed by and accumulate into parenting dimensions which together comprise general parenting styles and are informed by the broader family context (in line with family systems theory). When considering adolescent substance use as an outcome, these styles are unlikely to map onto the classical parenting styles exactly, and likely include pro- and anti-alcohol (or marijuana, or other substance use) attitudes which are likely also passed down intergenerationally. Analytically, incorporating longitudinal data at multiple timescales in both person- and variable-centered approaches to examining the interactive, combined role of multiple parenting behaviors and dimensions are likely to yield important information about how parenting co-develops with child biobehavioral risk. As reviewed above, there is a solid literature base examining parenting cross-sectionally under this conceptualization. However, to move this work forward, we must include not only mothers, but fathers and other caregivers in these models, as well as other members, relationships, and attributes of the family system, as well as by testing the mechanisms by which parenting influences operate which would be greatly aided by leveraging genetically informed designs.

The co-development of parenting and child biobehavioral risk is conceptualized as dynamic, and shaped both by parents, children, and the genetics and environments (including broader culture and context influences) that they do and do not share. As noted above, one way to incorporate this co-development of multiple layers of parenting with multiple aspects of child biobehavioral risk is though variable- and family centered data aggregation techniques applied to longitudinal data at the specific timescales they are expected to operate (e.g., moment-to-moment for parenting and child behaviors, in the mid-term like over months for parenting dimensions and slower biological and behavioral transitions like puberty and substance use uptake progression).

The mechanisms by which day-to-day interactions canalize or cement themselves into longer-term patterns of interaction and trajectories are conceptualized in terms of dynamical systems, whereby periods of transition introduce variability into the dyadic and family systems, which allows for shifts in longer-term trajectories. This perspective means that times of transitions are critical to leverage in intervention and prevention work as developmentally sensitive periods for reducing substance use risk. Analytically, a combination of time-varying effects models (Nilsson, 2016; Mak et al., 2020), latent cross-lagged panel models that separate within- and between-family variance, and other techniques for disaggregating between-family levels and within-family changes would provide insights into how these dyadic and family system dynamics unfold over time.

Ideally, studies using multiple timescales of measurement (e.g., measurement burst designs where data are collected on shorter time scales repeatedly across longer timescales) would be leveraged to understand the transactional development of child and parenting risk for substance use. Specifically, repeated measures on shorter time scales can be aggregated into dyadic process features (e.g., dyadic patterns of specific parenting behaviors and adolescent responses over days or weeks) that can then be modeled longitudinally over time. An additional analytic advancement critical to integrating dynamical systems theories in this model is to introduce transitions (e.g., not only adolescent transitions like schools or extracurricular involvement, but also family transitions to capture the core tenets of family systems theory, adolescent biological transitions like pubertal milestones, and adolescent behavioral transitions like substance use milestones) as “knots” in developmental trajectories in the longer-term developmental models (e.g., using methods stemming from piecewise growth curves). Doing so would allow for the timing (e.g., age, grade, stage of puberty) at children’s knot/transition points, as well as the individual/families’ slopes of change for shorter time-scale dyadic process features to be explicitly modeled to provide insights on how dyadic dynamic systems of substance-specific parenting behaviors are related to individual child trajectories of substance use risk.

## Conclusion

The proposed bioecological systems cascade model draws together several theoretical approaches (e.g., Bioecological, Dynamical Systems, Family Systems, Developmental Cascades) to illustrate the complex role that parenting plays in the development of adolescent substance use. There is already empirical support for many aspects of this model. Recent and future advancements in study design and analytic methods, some of which are delineated above, will allow for more robust testing of this complex developmental model. Embracing more complex models like the bioecological systems cascade model proposed here will help the field move toward a more realistic depiction of the variability and changes inherent in families and help move the field away from describing sample average patterns that dismiss the variability that exists within and across families over time. Using methods of data reduction that can map onto less frequently studied but meaningful theoretical concepts, such as quantifying the timing and types of transitions, variability at multiple timescales, and co-development of parenting and child biobehavioral risk provide rich avenues of future research into the role parents and families play in the development of adolescent substance use. As we move toward embracing complexity, it will be critical to include diverse samples, with respect to socioeconomic advantage, social context (including cultural factors), family structures, parents (e.g., genetic related and unrelated parents; mothers and fathers; same- and differing gendered co-parents; one through multi-parent families; other caregivers), and adolescents (e.g., gender diversity, neurodiversity, and adolescents’ cultural contexts). Doing so will enable us to understand similarities and differences in how, when, and which aspects of parenting help to shape adolescent risk for substance use.

Findings from tests of this model are expected to inform prevention and intervention strategies, particularly by identifying specific circumstances (e.g., substance use-specific parenting behaviors in specific family and cultural-context climates) at specific timepoints (e.g., developmental knots that are marked by transitions, the likely developmental timing of which may be predicted by measurable intergenerational factors) are most likely to produce canalized changes in the positive or negative direction. Insights from this model may also yield a better understanding of when different parenting behaviors can help or do more harm than good in terms of adolescent substance use risk. Parents of adolescents face the challenge of striking the right balance between autonomy-granting and protective behaviors. The right balance undoubtedly depends on the child’s characteristics, and the developmental, cultural, and family context in which the family finds themselves. While daunting, the many transitions faced by adolescents and parents allows for repeated opportunities to readjust interaction patterns and shape parent-adolescent trajectories in the most positive directions afforded by the family and cultural context. In order to understand the process of striking this balance, and in turn to help parents navigate this balance, it is important to embrace the complexity of the child-parent relationship in the context of the family, development, and larger cultural context to understand the role of parenting for the development of adolescent substance use. Given the availability of large, genetically informed, longitudinal samples, the increasing focus on diversifying samples, and advancements in our methodological tools, it is possible to do so.

## Data availability statement

The original contributions presented in the study are included in the article/supplementary material, further inquiries can be directed to the corresponding author.

## Author contributions

KM: Writing – original draft.
